# The Autonomic Coumel Triangle: A New Way to Define the Fascinating Relationship between Atrial Fibrillation and the Autonomic Nervous System

**DOI:** 10.3390/life13051139

**Published:** 2023-05-08

**Authors:** Marco Rebecchi, Francesca Fanisio, Fabio Rizzi, Alessandro Politano, Ermenegildo De Ruvo, Cinzia Crescenzi, Germana Panattoni, Marianna Squeglia, Annamaria Martino, Stefano Sasso, Paolo Golia, Giulia Pugliese, Sofia Del Gigante, Domenico Giamundo, Pietro Desimone, Domenico Grieco, Lucia De Luca, Ignazio Giordano, Francesco Barillà, Marco Alfonso Perrone, Leonardo Calò, Ferdinando Iellamo

**Affiliations:** 1 Division of Cardiology, PoliclinicoCasilino, 00169 Rome, Italy; marcorebecchi3@icloud.com (M.R.); fanisio.francesca@gmail.com (F.F.); fabio.rizzi@hotmail.it (F.R.); politano.alessandro@yahoo.it (A.P.); gildo_deruvo@icloud.com (E.D.R.); crescenzi.cinzia@gmail.com (C.C.); germana.panattoni@gmail.com (G.P.); marianna.sgueglia@gmail.com (M.S.); martinoannamaria@yahoo.it (A.M.); p.golia@hotmail.it (P.G.); dgrieco.polcas@eurosanita.it (D.G.); lucia.delucaep@gmail.com (L.D.L.); lcalo.polcas@eurosanita.it (L.C.); 2 Department of Systems Medicine, University Tor Vergata, 00133 Rome, Italy; stefanosasso95@libero.it (S.S.); giulia.pugliese90@hotmail.it (G.P.); delgigantesofia932@gmail.com (S.D.G.); jamundus20@libero.it (D.G.); pietro.desimone94@libero.it (P.D.); ignazio994@gmail.com (I.G.); brlfnc01@uniroma2.it (F.B.); 3 Department of Clinical Science and Translational Medicine, University Tor Vergata, 00133 Rome, Italy; marco.perrone@uniroma2.it

**Keywords:** atrial fibrillation, autonomic nervous system, sympathetic, parasympathetic, ion channels, pharmacological therapy, ablation techniques

## Abstract

Arrhythmogenic substrate, modulating factors, and triggering factors (the so-called Coumel’s triangle concept) play a primary role in atrial fibrillation (AF) pathophysiology. Several years have elapsed since Coumel and co-workers advanced the concept of the relevance of autonomic nervous system (ANS) influences on atrial cells’ electrophysiological characteristics. The ANS is not only associated with cardiac rhythm regulation but also exerts an important role in the triggering and maintenance of atrial fibrillation. This review aims to describe in detail the autonomic mechanisms involved in the pathophysiology of atrial fibrillation (AF), starting from the hypothesis of an “Autonomic Coumel Triangle” that stems from the condition of the fundamental role played by the ANS in all phases of the pathophysiology of AF. In this article, we provide updated information on the biomolecular mechanisms of the ANS role in Coumel’s triangle, with the molecular pathways of cardiac autonomic neurotransmission, both adrenergic and cholinergic, and the interplay between the ANS and cardiomyocytes’ action potential. The heterogeneity of the clinical spectrum of the ANS and AF, with the ANS playing a relevant role in situations that may promote the initiation and maintenance of AF, is highlighted. We also report on drug, biological, and gene therapy as well as interventional therapy. On the basis of the evidence reviewed, we propose that one should speak of an “Autonomic Coumel’s Triangle” instead of simply “Coumel’s Triangle”.

## 1. Introduction

Arrhythmogenic substrate, modulating factors, and triggering factors (the so-called Coumel’s triangle concept) play a primary role in atrial fibrillation (AF) pathophysiology. Several years have elapsed since Coumel et al. [1] advanced the concept of the relevance of autonomic nervous system (ANS) influences on atrial cells’ electrophysiological characteristics. The ANS is not only associated with cardiac rhythm regulation but also exerts an important role in the triggering and maintenance of atrial fibrillation. The heart is richly innervated by the autonomic system that can be located either inside the heart (intrinsic ANS) or in brainstem and cardiac preganglionic fibers (extrinsic ANS). Both intrinsic and extrinsic autonomic nervous system divisions are fundamental for cardiac function and arrhythmogenesis [2,3,4]. From a pathophysiological as well therapeutic perspective, the role of the epicardial autonomic ganglionated plexus (GP) in the initiation and maintenance of AF should be highlighted.

## 2. The Biomolecular Explanation of the Autonomic Nervous System Role in Coumel’s Triangle

Each of the myocardial sleeves of the pulmonary veins (PVs) is richly innervated by four of the left atrial GP [5]. These four GPs are localized in areas of fractioned atrial potentials, as widely described in the literature [6]. Po et al. demonstrated that the injection of acetylcholine into the GP can induce focal firing coming from adjacent pulmonary veins [7]. Moreover, in animal models of PV preparations with a surrounding left atrial myocardial tissue, local autonomic stimulation induces early afterdepolarizations (EADs) and bursts of triggered firing from the PVs [8]. This triggered activation was similar to the pattern recorded from the PVs in patients with paroxysmal AF [6]. It must be underlined that in autonomic nerve structures, there is a frequent co-localization of sympathetic and parasympathetic fibers making a complete selective radiofrequency catheter ablation extremely difficult. The spatial and functional interplay between sympathetic and parasympathetic fibers plays a relevant role in starting and maintaining the AF burden [9]; for example, sympathetic stimulation would promote cholinergic-mediated AF initiation [10].

### 2.1. Cardiac Autonomic Neurotransmission: Adrenergic and Cholinergic Molecular Pathways

**Figure 1:**
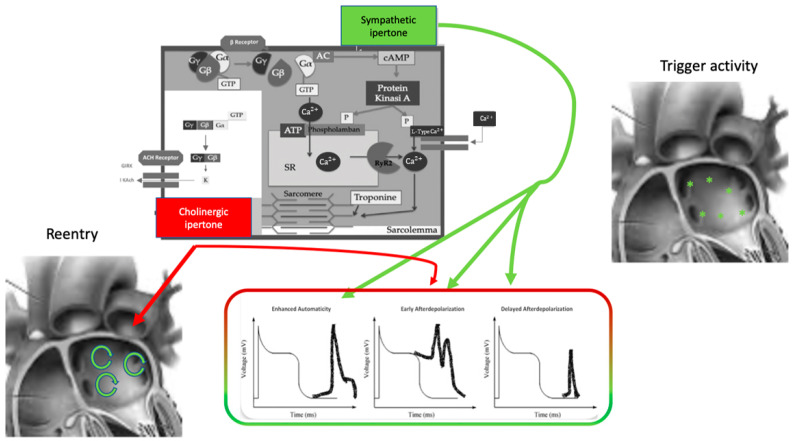
Autonomic nervous system and atrial fibrillation. Biomolecular mechanisms and the influence of the ANS on triggers and atrial reentry mechanisms. See text for explanation.

The effect of the autonomic nervous system on cardiomyocytes is mainly related to changes in ion channel functioning, leading to action potential length, conductive forces, and conduction velocity modifications (Figure 1). The extremely complex mechanism of neurotransmitter production, release, and reuptake is highly regulated [11].

Norepinephrine is the principal neurotransmitter of the sympathetic autonomic system: it is synthesized in neural cell bodies and stored in vesicles adjacent to nerve varicosities close to adrenergic receptors where they are released through a Ca^2+^-dependent process.

Norepinephrine binds to a variety of adrenergic receptors in cardiomyocytes and the precise, extremely complex mechanisms have been broadly described [12].

The β-adrenergic receptor is a member of the family of 7-transmembrane domain G protein-coupled receptors. It comprises three subtypes, β_1–3_, of which β_1_ receptors are the most involved in the pathogenesis of atrial arrhythmias. Norepinephrine’s link to the β-receptor leads to a free Gα_s_ subunit that activates adenylate cyclase and converts ATP to cAMP. cAMP is the primary β-adrenergic second messenger; it activates protein kinase A (PKA) that phosphorylates membrane proteins, including Ca^2+^-handling proteins, ion channels, and phospholamban (PLB). Moreover, epinephrine stimulation increases Ca^2+^ binding to calmodulin (CaM), activatesCa^2+^-/CaM-dependent kinase type II (CaMKII), inhibits the inward rectifier K^+^ current (I_K1_), and enhances the slow-delayed rectifier K^+ 2^ current (I_Ks_). These actions have led to increases in systolic Ca^2+^ transient and contraction strength [13].

Parasympathetic influence on cardiomyocytes depends on acetylcholine binding on the cholinergic receptor M2 type2 muscarinic subtype. M2-acetylcholine receptors are G-coupled, and they are formed by inhibitory G protein Gαi linked to the Gβγ subunit. Acetylcholine interaction with the M2receptor causes the dissociation of Gβγ subunits from Gαi with Gβγ activation of the ligand-gated K^+^ channel IKACh [14]. IKACh activation causes an outward K^+^ current that results in a decrease in action potential duration (APD). Moreover, heart rate and atrial electrophysiology regulation depends on G protein-coupled inwardly rectifying K^+^ channels (GIRK) associated with muscarinic and adenosine receptors [15]. GIRKs are located in the sinoatrial node (SAN), atria, atrio-ventricular node (AVN), and Purkinje fibers and lead to membrane hyperpolarization with a subsequent slower firing rate in the SAN. Acetylcholine binding to its muscarinic receptor influences the funny current (If), promoting a more negative action potential and lowering firing rate in the sinoatrial cells. As for intercellular mechanisms, acetylcholine can inhibit gap junction communication leading to contractility velocity reduction through the atrium [16].

### 2.2. Interplay between Autonomic Nervous System and Cardiomyocytes Action Potential

The autonomic nervous system promotes AF by focal or reentrant mechanisms (Figure 1) [17,18]. Adrenergic activation may promote focal activity by three main principal cellular mechanisms: enhanced automaticity, early afterdepolarization (EAD), or delayed afterdepolarization-associated triggered activity (DAD) [19]. Adrenergic activation causes increased automaticity through the prevention of spontaneous phase 4 depolarization which is promoted by If and inhibited by the IK1 current. Moreover, beta-adrenergic activation may raise focal activity through two principal cellular mechanisms: early afterdepolarization or delayed afterdepolarization-associated triggered activity [20]. Norepinephrine can lead to EAD in a setting of prolonged or shortened action potential duration (APD). β-adrenergic activation through PKA/CaMKII phosphorylation enhances plateau I_Ca_, leading to an increased phase 2 EAD likelihood, especially in individuals suffering from long-QT syndrome, in whom adrenergic augmentation of IKs is deficient [21]. Phase3-EAD is associated with increased Ca^2+^ transient and shortened APD: it is caused by the simultaneous activation of the sympathetic nervous system with increased Ca^2+^ and parasympathetic nervous system activation of IKAch. The simultaneous presence of short APD and increased Ca^2+^ transient leads to late-phase 3 EAD and trigger arrhythmias [8,22,23]. As a matter of fact, APD prolongation permits L-type Ca^2+^ channels to recover from inactivation, leading to an inward current, causing an EAD.

B-adrenergic stimulation alone can also lead to phase 2 EAD in a setting of prolonged APD by increasing the depolarizing currents (L-type Ca^2+^ current [ICa, L]) [19]. DAD results from the dysfunction of channel type 2 ryanodine receptor (RyR2) caused by the increased phosphorylation of Ca^2+^/calmodulin-dependent protein kinase II (CaMKII); it promotes SR Ca^2+^ release and Na^+^/Ca^2+^ exchanger (NCX) activation. All these processes produce a depolarizing transient inward current (Iti) that causes delayed afterdepolarization.

The molecular basis of maintaining reentry mechanisms is not clearly understood; however, in all conceptual models, functional reentry is promoted by atrial refractoriness [24]. Vagal stimulation abbreviates atrial refractoriness by increasing IKACh. In addition, atrial refractoriness abbreviation made by parasympathetic stimulation is characterized by a relevant regional variability that perpetuates the vagal AF-promoting effects. The role of increased refractoriness spatial heterogeneity is confirmed in animal studies in which flecainide terminated the vagus-related pro-arrhythmic effects on AF [25]. A very interesting observation was that, in an ion channel model, a reentry spiral wave induced by a sympathetic stimulation tended to organize and terminate immediately, whereas that induced by a vagal stimulation tended to be maintained in the presence of the refractoriness spatial dispersion. Therefore, this is the explanation as to why an adrenergic AF usually terminates spontaneously, while a vagal AF tends to be maintained [26].

**Figure 2:**
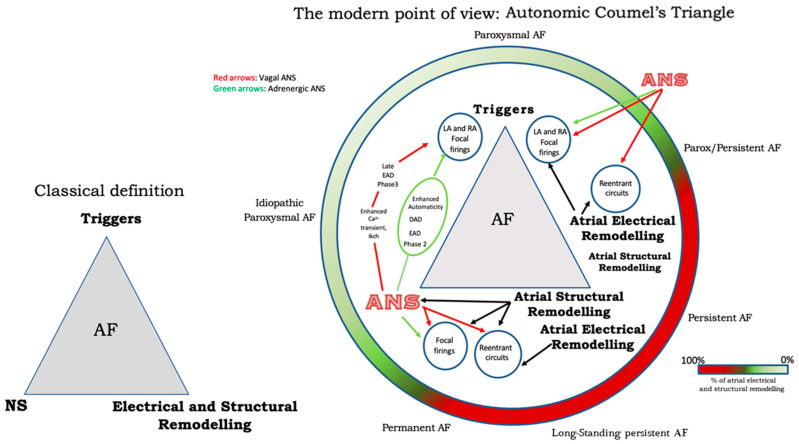
The Autonomic Coumel’s Triangle. See text for explanation.

Finally, Figure 2 illustrates a proposed point of view regarding the systemic role of the ANS in Coumel’s triangle. The hypothesis of an “Autonomic Coumel Triangle” stems from the condition of the fundamental role of ANS in all phases of the pathophysiological mechanism of AF. As we know, an increase in sympathetic tone favors trigger activity (e.g., ectopic activity from the pulmonary veins, but also from the right atrium) through three known mechanisms: enhanced automatism, EAD, and DAD. Together with sympathetic activation, vagal activity can contribute to triggering activity through the creation of a late-phase 3 EAD. This mechanism is very often present in paroxysmal AF in both healthy and young hearts. However, the role of ANS is also fundamental in forms of persistent AF and/or standing persistent AF where the substrate prevails. In these patients, the role of parasympathetic activity in favoring a spatially heterogeneous action potential and refractory period abbreviation that promotes the occurrence and maintenance of reentrant activity is of crucial importance. At the same time, sympathetic hyperactivity is able to contribute to arrhythmic substrate by promoting increased Ca^2+^/calmodulin binding and oxidative stress.

## 3. Autonomic Nervous System and Atrial Fibrillation: A Heterogeneous Clinical Spectrum

The ANS plays a relevant role in situations that may promote the initiation and maintenance of AF. Some patients show arrhythmic forms linked to vagal hyperactivity, e.g., after abundant meals due to gastric distension and/or gastro-esophageal reflux and after neuro-mediated syncope. This may also occur in bradycardic patients with nocturnal onset AF or forms of AF linked to obstructive sleep apnea (OSA), generally without structural cardiac changes. On the other hand, some patients show forms of AF that is triggered and sustained by the adrenergic nervous system, for example, AF in athletes during intense physical activity or in patients subjected to intense emotional stress.

In patients with the vagal type of AF, there is usually a smaller LA volume and better electrical properties. In contrast, in patients with the sympathetic type of AF, the LA substrate is worse, coexisting with non-PV triggers and recurrence [27].

Chronic pathologic states that affect the left and/or right heart and the pulmonary system such as heart failure and pulmonary hypertension predispose patients to AF [28].

A link between AF and gastro-enteric disorders has also been suggested [29,30]. In particular, an association between gastro-esophageal reflux disease (GERD) and esophagitis and AF has been proposed, given the close anatomical proximity between the esophagus and the left atrium. In this context, episodes of AF triggered by defecation, alcohol, or cold water ingestion and fatty food intake have frequently been reported in patients with GERD [31].

Gastro-esophageal reflux is further associated with an increase in vagal activity: the acid reflux may cause a local inflammation that may directly penetrate the esophageal wall which, in turn, stimulates the adjacent vagal nerves [32,33].

Studies in lone AF patients and in animal models of intermittent rapid atrial pacing and congestive heart failure have indicated that AF onset is associated with simultaneous sympathovagal activation rather than with an increase in vagal or sympathetic drive alone [34,35]. Additionally, the effect of vagal stimulation on atrial refractoriness is heterogeneous because of the distribution in parasympathetic nerve endings and/or Muscarin-2 heterogeneity receptor. In patients with GERD, the increased vagal activation creates an arrhythmogenic substrate for reentry circuits, thereby increasing susceptibility to AF [36,37]. Inflammation constitutes the basic link between gastrointestinal disturbances and the occurrence of AF, as occurs in inflammatory bowel disease (IBD) [38]. This suggestion would be supported by the presence of electrocardiographic P-wave dispersion, a risk factor for AF, in patients with IBD [39].

In addition, syncope is associated with AF, and they frequently occur together in an acute clinical scenario [40,41], even though AF rarely causes syncope.

Coumel [1,42] was the first to recognize bradycardia-induced AF and ascribed this to vagal stimulation. Attuel et al. [43] showed that atrial refractory periods shorten with the slowing of heart rate in patients with vagal-induced AF. This paradoxical finding linked with an increase in atrial dispersion of refractoriness is the mechanism for the vagal induction of AF. Vagally mediated AF usually occurs in the setting of structurally normal atria and the degree of refractory period dispersion created by the vagal shortening of atrial action potentials appears to be the major determinant of vagally mediated AF [42].

Several mechanisms have been advanced to explain the development of AF in patients with OSAS. These include hypoxia, structural atrial remodelling and, again, autonomic imbalance [44]. Stevenson et al. [45] hypothesized that hypoxia would contribute to alter cardiac conduction via the shortening of action potentials, prolongation, and heterogeneity of refractory periods, early afterdepolarizations, and increased PV firings. Severe intermittent hypoxemia, acidosis, and hypercapnia can result in sympathetic activation leading to HR and blood pressure (BP) elevation [46]. Conversely, hypoxemia in the setting of apnea can also trigger a vagal response [47] and the negative intrathoracic pressure stimulates the parasympathetic nervous system. The ANS imbalance may precipitate electrical changes in the atrium that predisposes individuals to AF [48]. Indeed, the neural activity of the left stellate ganglion has been shown to be enhanced in OSAS as compared to controls in a dog model and this was thought to accelerate LA neural remodelling and enhance the risk of AF [49]. Indeed, superior left ganglionic plexus ablation has been shown to suppress AF in a mouse model of chronic OSAS by inhibiting its sympathovagal hyperactivity [50]. Additionally, low-level vagus nerve stimulation was capable of suppressing acute AF in the same animal model [51].

The link between sport and AF should not be forgotten. In this context, the role of the ANS is fundamental and varies according to the type and intensity of the sporting activity. In fact, episodes of AF are well established in athletes with predominant vagal activity at rest and during low-intensity physical work. On the contrary, adrenergically mediated AF occurs in athletes presenting with high sympathetic activity during strenuous exercise [52].

## 4. From Pharmacological and Interventional Therapy to Future Perspectives

### 4.1. Drug, Biological, and Gene Therapy

Bearing in mind the crucial role of the ANS in the pathophysiology of AF, it should be possible to identify drugs affecting ANS for AF prevention and treatment.

Betablockers are highly effective in preventing AF recurrence after electrical cardioversion. Knowing the autonomic contribution to AF, it may then be possible to target therapy based on it. For instance, in patients undergoing cardiac surgery, for whom there is evidence of an important role of Ca-homeostasis abnormalities in post-operative AF, prophylactic betablockers are particularly effective in preventing post-operative AF [53].

Among the studies on drug therapy for vagally mediated AF, class I and class III antiarrhythmic drugs (AAD) are the most reported. Wang et al. [54] reported that flecainide terminated vagally mediated AF episodes in all of 16 dogs by increasing the atrial effective refractory period (ERP). Flecainide also increased the reentry wavelength while decreasing the number of functional reentry circuits resulting in a reduced propensity to vagally mediated AF [54].

Other old and new drugs do exist which are capable of terminating AF, each one with its own mechanism of action.

Pilsicainide is a new class IC antiarrhythmic drug that preferentially inhibits Na^+^ conductance with a consequent reduction in the maximal rate of increase in action potentials (V_max_). In a clinical study, Pilsicainide successfully terminated AF induced by vagal stimulation. It is likely that pilsicainide suppressed the intraatrial conduction at faster atrial rates to a crucial point, beyond which the propagation of activation would be impossible [55]. Disopyramide, a class IA antiarrhythmic drug, has a Na channel-blocking effect and is suggested in the treatment of vagally mediated AF, given its prominent vagolytic effect [56]. Propafenone, an IC class AAD, on the contrary, is not recommended in vagally mediated AF because of its coexisting beta-blocking effect that may facilitate AF during bradycardia [57].

Kondoh et al. [58] reported the effectiveness of MS-551, a class III AAD for VM-AF in six out of eight dogs in a canine myocardial infarction model. MS-551 inhibited IK and decreased the transient outward current (Ito) and the IK1 current. Sotalol has class III antiarrhythmic and beta-blocker effects; Yesil et al. [59] concluded that sotalol is more effective in treating adrenergic AF, but could also be effective in selected patients with vagally mediated atrial fibrillation without baseline bradycardia. Given the importance of ACh-gated potassium currents (IKACh) in AF, selective blockers are being developed in preclinical studies [60]. In addition, our group [24] emphasized the importance of IKACh inhibitors as a therapeutic target for vagally mediated AF, also deprived of toxicity and capacity of AP prolongation and neurological effect. Tertiapin was the first IKAch selective inhibitor tested in a canine model. Hashimoto et al. reported that tertiapin prolongs the effective refractory period (AERP) without affecting ventricular repolarization during vagal nerve stimulation and terminates AF with 100% efficacy [61].

Biological therapies targeting G proteins have been applied to modulate the AV nodal function and control the ventricular response in AF, as well as to prevent AF induction in a vagal model [62]. The modification of G protein subunit activity, by using viral or non-viral gene transfer methods, represents one of the most important targets of gene therapy. Viral vectors could be used to overexpress the G proteins, determining a reduction in AF rate secondary to AV conduction inhibition [63].

Finally, Botulinum toxin injection into epicardial fat pads rich in autonomic ganglia can temporarily suppress VM-AF inducibility for approximately 1 week in a canine model compared to a control before the effects wear off in week 2 and week 3. The underlying mechanism has been associated with parasympathetic autonomic modification by a ganglionic block that reduces AERP dispersion [64]. Several years later, a good result, in terms of reduction in AF recurrences at long-term follow-up, was also obtained by Botulinum injection into epicardial fat pads in patients undergoing coronary artery by-pass surgery [65].

### 4.2. From Cardiac to Non-Cardiac Neuromodulation Therapy

One of the most interesting findings as to the role played by the ANS in the occurrence of AF came from the observation that in patients with paroxysmal AF, during pulmonary vein isolation, there was greater efficacy of the procedure in those in whom the evocation of a vagal reflex was detected during radiofrequency application [3]. Topographical anatomy studies [66] showed that human intrinsic atrial cardiac epicardial ganglia form five main GPs, located at different sites of the atrial surface. It was later found that some of these ganglionic plexi are gateways for the sinoatrial and atrioventricular node function. In other words, they are considered as “integration centers” between extrinsic and intrinsic autonomic cardiac innervations [67]. Among these, the most representative are considered the anterior right GP (ARGP, located near the SA node), and inferior right GP (IRGP, at the junction of the inferior vena cava and atria).

Several studies have demonstrated the efficacy of the endocardial ablation of GPs in the left atrium (sometimes with a bi-atrial approach) in combination with pulmonary vein isolation. For several years, the vagal denervation scenario was characterized by a strong scientific debate as to whether a selective approach (i.e., administration of radiofrequencies to the areas of vagal reflex evocated during high-frequency stimulation) was better than an anatomical approach (that is, based on ablation of the anatomical sites of the ganglionic plexi) [68].

Our group has been firmly convinced for a longtime that concentrating (at least initially) the ganglionated plexianatomical ablation procedure in the right atrium is sufficient to achieve a satisfactory clinical outcome, in terms of reductions in AF recurrences [69]. Several observations led our group to propose the above type of approach in the right atrium: (a) first, anatomical studies showed an extensive distribution of ganglia in the right atrium [69] and (b) the presence of the so-called “third fat pad”, placed at the junction between the right atrium, the inferior vena cava, and between the latter and the aorta. The “third fat pad” is considered to be the entry point for vagal input to the GP, before innervating both the RA and LA. Finally, some GPs of the right atrium could remotely modulate the function of the GPs of the left atrium through anatomical interconnections [24].

Currently, this approach could be further refined through the introduction of the concept of “Cardioneuroablation”, i.e., an ablation method based on the identification (by means of electroanatomical mapping systems) of particular endocardial potential, expressions of ANS tissue interposition, in the context of atrial fibrillar myocardium [19].

The scenario of interventional therapy on the sympathetic–vagal imbalance in patients with AF is also supported by observations from non-direct cardiac neuromodulation [24]. First of all, Renal Sympathetic Denervation (RSD) would be effective in reducing adrenergic AF recurrences in patients suffering from resistant hypertension. Several investigations have shown that a combined approach, represented by pulmonary vein isolation plus RSD, significantly decreases AF recurrence when compared with pulmonary vein isolation alone.

## 5. Conclusions

The key role played by the ANS in the complex pathophysiological mechanisms leading to AF is well recognized. For a longtime, we used the terms vagal AF and adrenergic AF to describe the primary autonomic mechanism underlying AF. Very often, however, vagal and adrenergic hyperactivity can coexist in the same patient. These pathophysiological prerequisites constitute the basis for therapeutic treatment aimed at correcting triggering factors (e.g., OSAS, gastro-esophageal reflux, gastric hyperdistension, alcohol abuse) and neuromodulation by vagal and adrenergic AF, that is, cardiac (cardioneuroablation) and non-cardiac neuromodulation.

The current deepened knowledge of Coumel’s triangle and of ANS physiology allows us to state that the ANS is capable of influencing both the trigger and the substrate of AF, playing a fundamental role in all clinical forms of this widely diffused arrhythmia (both in its paroxysmal and persistent forms). These considerations prompt us to suggest that one should speak of an “Autonomic Coumel’s Triangle” instead of simply “Coumel’s Triangle”.

## Data Availability

Not applicable.
